# A biocompatible macromolecular two-photon initiator based on hyaluronan†
†Electronic supplementary information (ESI) available. See DOI: 10.1039/c6py01787h



**DOI:** 10.1039/c6py01787h

**Published:** 2016-11-29

**Authors:** Maximilian Tromayer, Peter Gruber, Marica Markovic, Arnulf Rosspeintner, Eric Vauthey, Heinz Redl, Aleksandr Ovsianikov, Robert Liska

**Affiliations:** a Institute of Applied Synthetic Chemistry , TU Wien (Technische Universitaet Wien) , Getreidemarkt 9/163/MC , 1060 Vienna , Austria; b Institute of Materials Science and Technology , TU Wien (Technische Universitaet Wien) , Getreidemarkt 9/308 , 1060 Vienna , Austria; c Physical Chemistry Department , Sciences II , University of Geneva , 30 Quai Ernest Ansermet , CH-1211 Geneva 4 , Switzerland; d Ludwig Boltzmann Institute - Experimental and Clinical Traumatology , Donaueschingenstraße 13 , 1200 Vienna , Austria; e Austrian Cluster for Tissue Regeneration , Austria

## Abstract

The possibility of the direct encapsulation of living cells *via* two-photon induced photopolymerization enables the microfabrication of hydrogel scaffolds with high initial cell loadings and intimate matrix–cell contact. While highly efficient water-soluble two-photon initiators based on benzylidene ketone dyes have been developed, they exhibit considerable cyto- and phototoxicity. To address the problem of photoinitiator migration from the extracellular matrix into the cytosol, a two-photon initiator bound to a polymeric hyaluronan backbone (**HAPI**) was synthesized in this work. **HAPI** exhibited a distinct improvement of cytocompatibility compared to a reference two-photon initiator. Basic photophysical investigations were performed to characterize the absorption and fluorescence behavior of **HAPI**. Laser scanning microscopy was used to visualize and confirm the hindered transmembrane migration behavior of **HAPI**. The performance of **HAPI** was tested in two-photon polymerization at exceedingly high printing speeds of 100 mm s^–1^ producing gelatin-based complex 3D hydrogel scaffolds with a water content of 85%. The photodamage of the structuring process was low and viable MC3T3 cells embedded in the gel were monitored for several days after structuring.

## Abstract 2

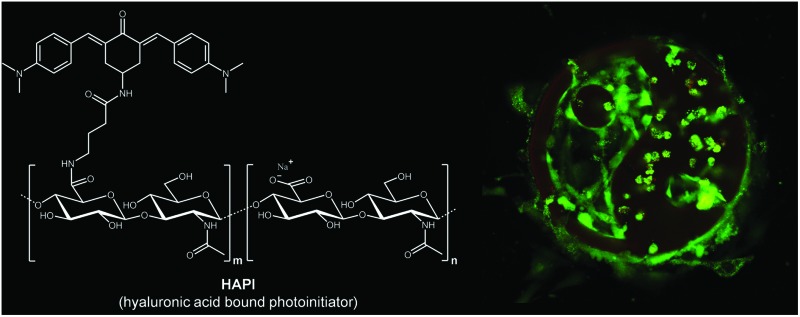
Binding a two-photon-initiator to hyaluronan hinders trans-membrane migration into cells and reduces cyto- and phototoxicity, enhancing biocompatibility.

## Introduction

The process of two-photon induced polymerization (2PP) has attracted considerable interest because it enables 3D printing with a resolution in the sub-micrometer range. Parts containing ultra-small features like photonic crystals, cantilevers, optical waveguides and microelectronic components may thus be produced.^1^ Furthermore 2PP can be employed to fabricate 3D scaffolds as a structural support for cell growth in tissue engineering.^2^ This is of great interest because 2D matrices used in traditional cell culture systems do not accurately reproduce the cells’ natural environment and lead to significant differences in the structure, function or physiology compared to living tissue. Various studies^3,4^ have investigated and demonstrated that 3D-matrix adhesions enhanced cellular functional activities compared to 2D adhesions. Bokhari *et al.* have shown that HepG2 hepatocytes grown on 3D polystyrene scaffolds are less susceptible to certain toxicological challenges than those grown in 2D.^3^

Usually such scaffolds are pre-fabricated with large enough pores and seeded with cells on the surface, which then migrate inside the scaffold, attach within the pores and proliferate there. Another popular approach is the encapsulation of cells within hydrogels, which can be cross-linked by different means, including photopolymerization. Cell encapsulation provides the advantages of high initial cell loading and more intimate cell–matrix contact, similar to that of the natural extracellular matrix (ECM).

Since biological tissue is relatively transparent at the laser wavelengths used for 2PP, arbitrary 3D structures can be created deep within aqueous media. Thus 2PP can in principle be employed to encapsulate living cells within 3D hydrogel structures by performing 2PP around them.^5^

Recently several specialized water-soluble two photon initiators (2PIs) have been developed and tested for their efficiency in the microfabrication of 3D hydrogel structures.^6^ While they possess a relatively high two-photon absorption (2PA) cross section compared to commercial water-soluble one-photon initiators like Irgacure 2959, and thus allow for structuring at high printing speeds and low laser intensities, there is still a need for novel initiators with improved cytocompatibility. Besides the mere cytotoxicity in darkness, the occurrence of additional phototoxicity upon irradiation is an important concern with PIs designed for biological applications.

The aforementioned phototoxicity can either be mediated by the active species that are generated from the PI itself, *i.e.* free radicals in the case of initiators for radical photopolymerization, or by other species that originate from secondary pathways. Among the non-radiative processes that lead to the relaxation of the involved excited triplet states of the PI, quenching by molecular oxygen under the formation of singlet oxygen (SO) and as a further consequence other reactive oxygen species (ROS) play an important role. Oxygen-activated species such as superoxide anions, hydroxyl radicals and singlet oxygen are by-products of oxygen-dependent reactions and have a wide potential for causing cell damage. Among these chemical entities, singlet oxygen is one of the most reactive, capable of damaging cells and tissues. Besides its relatively long lifetime in solution, from micro- to milliseconds, singlet oxygen behaves as a strong electrophile in solution and reacts avidly with molecules possessing regions of high electron density. The oxidative damage of cells mediated by singlet oxygen is common and DNA, proteins and lipids are all at risk.^7^ In fact, this is also made use of for medical purposes in photodynamic therapy.^8^ In the case of the encapsulation of living cells *via* 2PP using water soluble 2PIs, it is hypothesized that due to their size and low molecular weight, they are able to pass the cytoplasmic membrane and migrate inside the cells. Since the cells are highly transparent for the lasers used in 2PP, the 2PI molecules are excited within the cytoplasm and the generated radicals and ROS may damage vital structures inside the cells. Thus, limiting the diffusion of 2PIs through the cell membrane might be an efficient strategy to reduce the overall photodamage to the cells and tackle the problem of phototoxicity of the 2PP structuring process.

A promising approach designing molecules to realize this is increasing the size and molecular weight of the 2PI by modifying a polymeric backbone, covalently attaching several units of a suitable derivative based on a previously examined efficient low molecular weight 2PI.

Hyaluronan (HA) was chosen as a backbone for novel polymeric 2PIs in this present work. HA is a natural and vital part of the ECM ^9^ and is becoming increasingly important as a building block for the creation of new bio-materials with utility in tissue engineering and regenerative medicine.^10–12^ Furthermore there are suitable methods for chemical modification *e.g.* amidation,^13,14^ and it is a highly negatively charged polycarboxylate, which poses an additional hindrance to migration through the cytoplasmic membrane which also bears a net negative charge.^15^

Two recent publications describe the synthesis and application of several efficient 2PIs based on cyclic dibenzylidene ketones – the first one^16^ focuses on 2PIs intended for polymerizable formulations based on organic solvents and resins, while the second one^6^ discusses derivatives bearing carboxyl-groups linked to their amino donor-functionalities leading to water-solubility and thus extending the range of application to hydrogel-based materials. Since the water solubility is already provided by the polycarboxylate HA-backbone and additional carboxyl groups in the 2PIs would only make the synthesis and purification more difficult, the 2PI component should bear simple alkylamino-groups that provide good organo-solubility until the last step of coupling to HA.

It has been reported in the literature that the quantum yield of singlet oxygen production from excited cyclic dibenzylidene ketones depends strongly on the size of the central ring, decreasing drastically for larger ring sizes in a series of cyclobutanone, cyclopentanone and cyclohexanone based derivatives.^17^

**Fig. 1:**
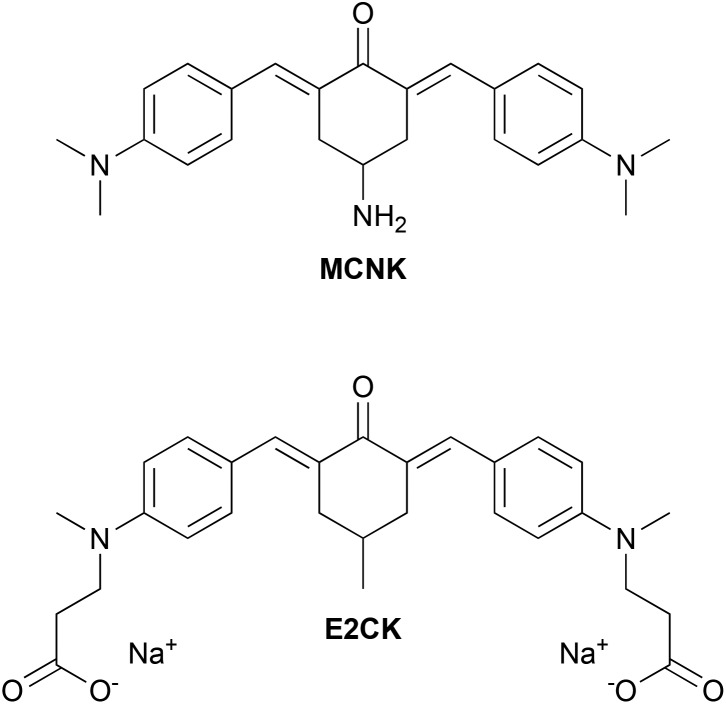
Precursor **MCNK** and reference **E2CK**, an efficient low molecular water-soluble 2PI.

Because of the prospect of lower phototoxicity for a six-membered central ring, the high initiation efficiency previously reported for similar derivatives,^6,16^ several successful reports of amidations of HA in the literature using 1,1′-carbonyldiimidazole (CDI)^14,18–20^ as well as commercial availability of suitable starting materials, the 2PI in this work was based on an amino substituted cyclohexanone. Fig. 1 shows the basic structure of the amino-cyclohexanone 2PI **MCNK** prepared in this work, and the previously published^6^ cyclohexanone based reference 2PI **E2CK**.

After linking **MCNK** to HA, the complete polymer-bound 2PI was characterized by various spectroscopic methods, tested for cytotoxicity in darkness and finally applied in the 2PP encapsulation of living cells.

## Experimental

### Materials and methods

#### Chemicals

Hyaluronan (hyaluronic acid sodium salt from *Streptococcus equi*, bacterial glycosaminoglycan polysaccharide), (4-oxocyclohexyl)carbamic acid *tert*-butyl ester (Boc-CNK), 4-(*tert*-butoxycarbonylamino)butyric acid (Boc-GABA-OH), 4-dimethylamino benzaldehyde and methyl-β-cyclodextrin (MBCD, BioReagent grade, 1.5–2.1 methyl per mol glucose) were purchased from Sigma-Aldrich and used without further purification. Solvents and other reagents were purchased from Sigma Aldrich, Fluka, Merck and Riedel-de Haen and were either used without further purification or dried and purified by standard laboratory methods.

#### Mode of practice for photosensitive compounds

The preparation and analysis of the photosensitive compounds and formulations was conducted in an orange light lab. The windows and fluorescent lamps were covered with a foil filter or filter coatings so that light with a wavelength <520 nm was cut off.

#### Melting point (mp), HR-MS, pH, gel permeation chromatography analyses

Melting points were measured with the aid of an automated melting point system (SRS OptiMelt MPA100). An Agilent 6230 LC TOFMS mass spectrometer equipped with an Agilent Dual AJS ESI source was used for HR-MS analysis. pH-Values were determined with a WTW pH 340i pocket meter. Molecular weights (*M*_n_) of HA before and after acidic degradation were determined by gel permeation chromatography using a Viscotek GPCmax VE 2001 with a VE 3580 RI detector calibrated with Shodex P-82 pullulan standards.

#### Nuclear magnetic resonance (NMR) spectroscopy

^1^H-NMR (200 MHz) and ^13^C-NMR (50 MHz) spectra were recorded with a BRUKER AC-E 200 FT-NMR-spectrometer. The chemical shift (s = singlet, bs = broad singlet, d = doublet, t = triplet, m = multiplet) is displayed in ppm using the non-deuterated solvent as the internal standard. Solvents with a grade of deuteration of at least 99.5% were used and purchased from EURISOTOP.

### Syntheses of precursors and the polymer-based two-photon-initiator (**HAPI**)

#### Synthesis of (3*E*,5*E*)-*N*-[3,5-bis[[4-(dimethylamino)phenyl]methylene]-4-oxocyclohexyl]carbamic acid 1,1-dimethylethyl ester (**Boc-MCNK**)

To a stirred solution of Boc-CNK (715 mg, 3.35 mmol) in ethanol (5 mL), a solution of 4-(dimethylamino)benzaldehyde (1 g, 6.70 mmol) and potassium hydroxide (188 mg, 3.35 mmol) in ethanol (5 mL) was added. The reaction mixture was heated to 60 °C and stirred for 18 h. After cooling, the resulting orange precipitate was recovered by suction filtration, washed with cold ethanol and dried *in vacuo*.

Yield: 1.12 g (70% of theory).

Mp: 219–222 °C.

HR-MS *m*/*z*: [M + H]^+^ calculated for C_29_H_38_N_3_O_3_ 476.2908; found 476.2932.

^1^H NMR (200 MHz, CDCl_3_): *δ*(ppm) = 7.88 (2H, s), 7.42 (4H, d, *J* = 8.9 Hz), 6.69 (4H, d, *J* = 8.9 Hz), 4.58–4.90 (1H, m), 4.09 (1H, bs), 2.72–3.25 (16H, m), 1.37 (9H, s).

^13^C NMR (50 MHz, CDCl_3_): *δ*(ppm) = 188.3, 155.1, 150.6, 139.9, 132.6, 128.1, 123.6, 111.6, 79.3, 45.2, 40.1, 34.4, 28.4.

#### Synthesis of (2*E*,6*E*)-4-amino-2,6-bis[[4-(dimethylamino)phenyl]methylene]cyclohexanone (**MCNK**)

Phosphoric acid (85% aqueous solution, 2 mL) was added to a suspension of **Boc-MCNK** (875 mg, 1.84 mmol) in dichloromethane (7.5 mL) and stirred vigorously to ensure constant mixing of the phases. After 3 h of stirring at ambient temperature, the solids had dissolved resulting in an almost colorless organic phase and a bluish grey aqueous phase. Deionized water (25 mL) was added, the reaction mixture cooled to 0 °C and sodium hydroxide (50% aqueous solution) was added drop-wise to bring the aqueous phase to pH ∼ 9. The aqueous phase was extracted with dichloromethane (3 × 75 mL), adding more water as necessary to dissolve the remaining solid phosphate salts. The combined organic layers were dried over Na_2_SO_4_ and stripped of the solvent *in vacuo*, resulting in orange flakes.

Yield: 656 mg (95% of theory).

Mp: 158–160 °C.

HR-MS *m*/*z*: [M + H]^+^ calculated for C_24_H_30_N_3_O 376.2383; found 376.2394.

^1^H NMR (200 MHz, CDCl_3_): *δ*(ppm) = 7.82 (2H, s), 7.43 (4H, d, *J* = 9.0 Hz), 6.69 (4H, d, *J* = 9.0 Hz), 3.10–3.30 (3H, m), 3.00 (12H, s), 2.55–2.83 (2H, m), 1.39 (2H, s).

^13^C NMR (50 MHz, CDCl_3_): *δ*(ppm) = 188.8, 150.5, 138.5, 132.5, 129.6, 123.9, 111.6, 47.0, 40.1, 38.1.

#### Synthesis of (3*E*,5*E*)-*N*-[4-[[3,5-bis[[4-(dimethylamino)phenyl]methylene]-4-oxocyclohexyl]amino]-4-oxobutyl]carbamic acid 1,1-dimethylethyl ester (**Boc-MGABA**)

Boc-GABA-OH (300 mg, 1.48 mmol) and CDI (239 mg, 1.48 mmol) were dissolved in anhydrous dichloromethane (3 mL) and stirred for 20 min after cessation of the initial gas formation. A suspension of **MCNK** (556 mg, 1.48 mmol) in dichloromethane (10 mL) was added and the resulting mixture was stirred for 18 h. After removal of the solvent *in vacuo*, the solid residue was recrystallized from ethyl acetate.

Yield: 830 mg (79% of theory).

Mp: decomposition >250 °C.

HR-MS *m*/*z*: [M + H]^+^ calculated for C_33_H_45_N_4_O_4_ 561.3435; found 561.3461.

^1^H NMR (200 MHz, CDCl_3_): *δ*(ppm) = 7.85 (2H, s), 7.37 (4H, d, *J* = 8.9 Hz), 6.64 (4H, d, *J* = 8.9 Hz), 6.49 (1H, d, *J* = 7.0 Hz), 4.86 (1H, bs), 4.25–4.49 (1H, m), 2.90–3.22 (18H, m), 2.13 (2H, t, *J* = 7.0 Hz), 1.70 (2H, quin, *J* = 6.8 Hz), 1.40 (9H, s).

^13^C NMR (50 MHz, CDCl_3_): *δ*(ppm) = 188.3, 172.1, 156.3, 150.6, 140.1, 132.7, 127.9, 123.5, 111.7, 79.1, 44.2, 40.1, 39.7, 33.9, 33.6, 28.4, 26.2.

#### Synthesis of (3*E*,5*E*)-4-amino-*N*-[3,5-bis[[4-(dimethylamino)phenyl]methylene]-4-oxocyclohexyl]butanamide (**MGABA**)

The deprotection of **Boc-MGABA** to obtain **MGABA** was carried out in analogy to the synthesis of **MCNK**.

Yield 94%.

Mp: 146–148 °C.

HR-MS *m*/*z*: [M + H]^+^ calculated for C_28_H_37_N_4_O_2_ 461.2911; found 461.2919.

^1^H NMR (200 MHz, CDCl_3_): *δ*(ppm) = 7.85 (2H, s), 7.36 (4H, d, *J* = 8.8 Hz), 6.74 (1H, d, *J* = 7.8 Hz), 6.63 (4H, d, *J* = 8.8 Hz), 4.27–4.48 (1H, m, *J* = 4.7 and 4.7 and 7.8 Hz), 3.06 (4H, d, *J* = 4.7 Hz), 2.97 (12H, s), 2.57 (2H, t, *J* = 7.0 Hz), 2.14 (2H, t, *J* = 7.2 Hz), 1.62 (2H, quin, *J* = 7.0 Hz), 1.31 (2H, s).

^13^C NMR (50 MHz, CDCl_3_): *δ*(ppm) = 188.3, 172.5, 150.6, 140.1, 132.7, 127.9, 123.4, 111.6, 43.8, 41.4, 40.0, 34.2, 33.7, 29.0.

#### Synthesis of hyaluronan-based photoinitiator (**HAPI**)

For low molecular weight **HAPI**, sodium hyaluronate (1.5 g, 1.6 MDa) was degraded by dissolving in deionized water (150 mL), adjusting to pH 1.00 with conc. HCl and by mechanical stirring at 60 °C for 24 h. After cooling down to room temperature and adjusting to pH 7.00–7.05 with TBA-OH (1 M in MeOH), the reaction mixture was stirred for 1 h and the white precipitate that formed was removed by centrifugation. The resulting clear solution was dialyzed against deionized water to remove small oligomers and excess TBA-Cl. Freeze-drying afforded 1.22 g tetrabutyl ammonium hyaluronate (TBA-HA) as a white fibrous solid.

An aliquot of TBA-HA (125 mg, 200 μmol HA repetition units) was dissolved in dry DMSO (12 mL) under an argon atmosphere. After the addition of CDI (9.8 mg, 60 μmol) and methanesulfonic acid (1.46 mg, 15 μmol) stirring was continued for 18 h, then **MGABA** (55.6 mg, 120 μmol) was added and the clear orange solution was stirred for another 72 h. Brine (1.2 mL) was added dropwise to the reaction mixture and after 2 h of stirring at ambient temperature, acetone (35 mL) was added. The resulting orange precipitate was separated by centrifugation and washed with acetone (3 × 45 mL) by stirring vigorously and subsequent centrifugation. The precipitate was then dissolved in deionized water (25 mL), the resulting red solution dialyzed against deionized water and freeze-dried to obtain **HAPI** as a bright orange fibrous solid.

Yields: degradation step – 1.22 g (81% of theory), modification step – 83 mg (95% of theory).

*M*_n_ (GPC of TBA-HA, 24 h degradation time): 50 kDa.

^1^H NMR (200 MHz, D_2_O) of **HAPI**: *δ*(ppm) = 7.57 (0.19H [varies with DS], bs), 7.01–7.45 (0.40H [varies with DS], m), 6.28–6.86 (0.39H [varies with DS], m), 4.19–4.64 (2H, m), 3.17–4.02 (10H, m), 2.52–3.03 (1.70 H [varies with DS], m), 1.92 (3H, bs).

### Photophysics

Absorption spectra were recorded on a Cary 50 absorption spectrometer. Fluorescence spectra were recorded on a Jobin Yvon FluoroMax-4. The fluorescence spectra were corrected for the wavelength dependent sensitivity of the apparatus using a set of secondary emissive standards.^21^ The samples were measured in PBS-buffer (Roti-Cell, Roth). Emission quantum yields were determined using Rhodamine 6G in methanol as the reference (*φ*_r_ = 0.95) according to
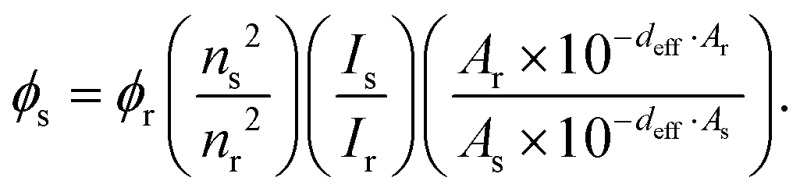


Here, *n*_*x*_, *I*_*x*_ and *A*_*x*_ are the refractive index, integrated emission intensity and absorbance at the excitation wavelength of *x*, with *x* being either the sample or reference. For the samples with quantum yields below 1%, the spectra were fitted with a lognorm function and the integral of the latter was used as *I*_s_.

Time resolved fluorescence experiments with an instrument response function of 200 ps were performed using a home-built single photon counting set-up, with excitation at 470 nm (LDH-P-C 470, PicoQuant), described in ref. 22.

Two-photon cross sections were determined *via* two-photon excitation spectra using a set-up similar to the one described in ref. 23 and detailed in ref. 24.

The two-photon cross section at a given wavelength, *δ*_s_^(2)^(*λ*), was calculated as follows^23^ 
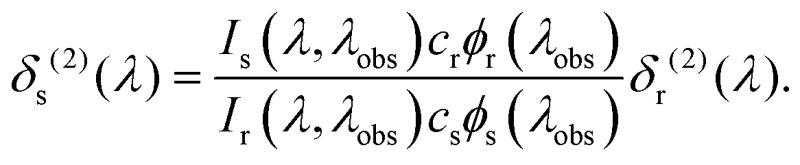


Here *I*_*x*_(*λ*,*λ*_obs_) is the (two-photon induced) fluorescence intensity at excitation wavelength *λ* and observation wavelength *λ*_obs_ for either sample or reference (*x* ∈ {s,r}). *c*_*x*_ and *φ*_*x*_(*λ*_obs_) are the concentration and differential fluorescence quantum yield (at the observation wavelength) of the sample and reference. Rhodamine 6G in methanol was used as the reference for determining the absolute two-photon cross sections, while Coumarin 120 in ethanol, Coumarin 153 in methanol and LDS 698 in chloroform, were additionally used for assigning the bandshape.^25^

### Cell culture

Mouse calvaria-derived preosteoblast cells (MC3T3-E1 Subclone 4) were obtained from ATCC-LGC Standards. MC3T3 were expanded in Alpha Minimum Essential Medium (αMEM) with ribonucleases, deoxyribonucleases, 2 mM l-glutamine, without ascorbic acid (Gibco), supplemented with 10% fetal bovine serum (Sigma) and 1% of 10 000 U mL^–1^ penicillin/streptomycin (Lonza). The cells were cultivated in an incubator in a humid atmosphere with 5% carbon dioxide at 37 °C. The medium was refreshed every second day.

### Evaluation of photoinitiator cytocompatibility

To evaluate the cytocompatibility of photoinitiators, PrestoBlue Cell Viability Reagent (Life Technologies) was used. For these tests 96-well plates were seeded with 5000 cells per well and incubated overnight for cells to attach. The cells were incubated with 100 μL of different dilutions of 2.0, 1.0 and 0.5 mM solutions of **HAPI** and **E2CK** for comparison. The procedure was performed under red light (620 nm LED) because of the light sensitivity and potential additional phototoxicity of the 2PIs. After 5 h of incubation with 2PIs, the culture medium was exchanged twice to remove 2PI residues and cell viability was evaluated. The resazurin-based reagent PrestoBlue was diluted 1 : 10 with the medium and 100 μL were applied per well and incubated for 1 hour. Because of the reducing environment of viable cells, this reagent is transformed and turns red, becoming highly fluorescent. The fluorescence was measured with a plate reader (Synergy BioTek, excitation 560 nm, emission 590 nm). After correction for background fluorescence, the results of the cells exposed to different concentrations of the photoinitiator were compared to each other and to the controls (non-stimulated cells). It was assumed that the metabolic activity of the control not exposed to photoinitiators is 100%. A statistical evaluation of data was performed using software packages IBM SPSS Statistic 22 (SPSS Inc., Chicago, IL) and Excel 2013 (Microsoft Office). Results are presented as the average of repeated measurements ± standard deviation (SD). After verifying the normal distribution and homogeneity of variance, a one-way analysis of variance was used to compare means of the samples. If a significant difference was observed (*p* < 0.05), Bonferroni *post hoc* multiple comparison tests were performed to detect a significant difference between the samples.

### Evaluation of 2PI transmembrane migration

MC3T3 cells were cultured in μ-dishes (35 mm diameter with glass bottom, high version, Ibidi GmbH, Martinsried, Germany) until the glass bottom was covered with a non-confluent monolayer of cells and then exposed to either **E2CK** or **HAPI** (as 0.1 mM solutions in PBS). After 5 min of incubation time, the autofluorescence of the 2PIs was visualized by laser scanning microscopy (Zeiss Axio Observer Z1 with an LSM 700 unit including an objective Zeiss EC Plan Neofluar 20×/0.5, ZEN11 software for evaluation) using the 488 nm laser for excitation.

### Two-photon-polymerization (2PP) printing and assay of encapsulated cells

The details of the 2PP microfabrication setup were reported previously.^5^ For the present work a femtosecond laser oscillator (MaiTai DeepSee by Spectra Physics) delivering 70 fs pulses at around 800 nm was used. The beam was focused into the sample with a water-immersion microscopy objective (32×/0.85).

Methacrylamide-modified gelatin (Gel-MOD) with a degree of substitution of 72% used in this experiment was prepared in accordance with a previously reported protocol.^26^ Structures were written in a solution of 15% Gel-MOD in αMEM containing 1 mM **HAPI**, 10 mM MBCD and a cell density of 10 million cells per 1 mL. The cell containing hydrogel precursor solution was applied to μ-dishes (35 mm diameter with a glass bottom, high version, Ibidi GmbH, Martinsried, Germany) where the glass slide had been functionalized with methacrylate groups by cleaning and activating with a 4 : 1 mixture of conc. H_2_SO_4_ and H_2_O_2_ (30% in water), and then using 3-(trimethoxysilyl)propyl methacrylate (Sigma Aldrich) according to the literature.^27^ The yin–yang structures were 2PP-fabricated starting directly on the glass surface to ensure proper adhesion *via* covalent bonding between the methacrylated glass surface and the crosslinked hydrogel, operating the 2PP system at the following parameters: laser power after objective 60 mW, scanning speed 100 mm s^–1^, hatch 0.5 μm, layer spacing 0.5 μm. The excess hydrogel precursor solution was removed after 2PP structuring by replacing the supernatant solution above the structures three times with fresh αMEM, and incubating for 1 h at 37 °C in-between exchanges. The cells were stained by calcein-AM and propidium iodide (Life Technologies) as previously described,^28^ 24 h and 5 days after 2PP structuring. Hydrogel constructs and encapsulated cells were visualized by laser scanning microscopy using the 488 nm and 555 nm lasers for excitation (Zeiss LSM 700 and ZEN11 software for evaluation).

## Results and discussion

### Synthesis and characterization

Classical aldol condensation reactions under alkaline catalysis are a powerful and cost-efficient tool to build 2PI chromophore systems.^16^ Since primary amines (necessary for coupling to HA) are reactive towards aldehydes and ketones, the prevention of unwanted side-reactions during storage or the aldol-condensation reaction requires the use of a protective group. Thus (4-oxocyclohexyl)carbamic acid *tert*-butyl ester (**Boc-CNK**) was used as a starting material, and condensed with 4-(dimethylamino)benzaldehyde. This forms a dibenzylidene bischalcone of the quadrupolar D–π-A–π-D structure with dimethylamino-groups as strong electronic donors (D) and the carbonyl group as the acceptor (A), promoting a high 2PA cross section.^16^ The reactants are heated in ethanol in the presence of potassium hydroxide, leading to the precipitation of the protected 2PI **Boc-MCNK** with a yield of 70%.

The deprotection was performed under mild conditions^29^ by stirring a suspension of **Boc-MCNK** in DCM vigorously with aqueous phosphoric acid and afforded the deprotected 2PI component **MCNK** with a yield of 95%.

First coupling attempts between HA and **MCNK** achieved only low degrees of substitution (DS) of about 5% (determined from ^1^H-NMR measurements by comparing the integrals of the aromatic protons of **MCNK** with the *N*-acetyl protons of HA), presumably because of the steric hindrance of the amino group on the cyclohexanone ring. Thus **MCNK** was amidated *via* a CDI based method^30^ by first reacting 4-(*tert*-butoxycarbonylamino)butyric acid (Boc-GABA-OH) with CDI to generate an activated carboxyl derivative, then adding freebase **MCNK** to form an amide bond and obtain **Boc-MGABA** (79% yield). Subsequent deprotection by aqueous phosphoric acid^29^ afforded the 2PI component **MGABA** (94% yield), providing a sterically less hindered amino group for attachment to HA.

**Fig. 2:**
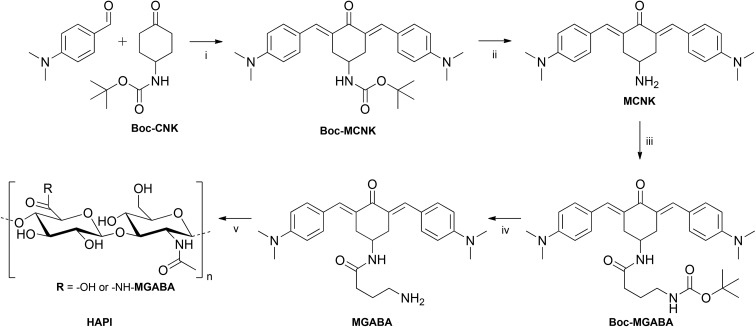
Synthetic pathway to **HAPI**. Conditions (i) KOH, EtOH; (ii) aq. H_3_PO_4_, DCM; (iii) CDI, Boc-GABA-OH, DCM, (iv) aq. H_3_PO_4_, DCM; (v) TBA-hyaluronan, CDI, MeSO_3_OH, DMSO.

For the final coupling step^14,18–20^ to generate the polymeric hyaluronan-based photoinitiator (**HAPI**), commercial sodium hyaluronate with an average molar weight of 1.6 MDa was first converted to DMSO soluble tetrabutylammonium hyaluronate (TBA-HA) *via* acidification with an ion exchanger resin and subsequent neutralization with TBA-OH solution. Then a part of the carboxylate groups (the residual unsubstituted groups ensuring water solubility) was activated *via* reaction with CDI, and subsequently **MGABA** was added to form the polymeric 2PI **HAPI**. The reaction was quenched with excess sodium chloride solution and **HAPI** was precipitated and washed with acetone to remove unreacted **MGABA**. Removal of the low molecular weight HA as well as cytotoxic TBA-cations by dialysis and subsequent freeze-drying afforded ready to use **HAPI**. A schematic representation of the whole reaction sequence is depicted in Fig. 2.

Preliminary coupling experiments between HA and **MGABA** achieved DS up to 10%, justifying the choice of the sterically more accessible amino group in comparison to **MCNK**. While **HAPI** prepared in this fashion was sufficiently water-soluble as a sodium salt, it could not be processed in 2PP structuring since a precipitation from the solution upon the addition of crosslinkable macromers like a modified gelatin (GelMod)^5,26^ occurred. As the first approach to improve the compatibility of **HAPI** with the macromers, the **HAPI** solubility was increased by using HA with a decreased molecular weight. While high molecular weight HA maintains homeostasis and potentially down-regulates inflammation, the generation of low molecular weight HA may act as an endogenous signal – likely mediated by cell surface receptors such as CD44 and TLR-4.^31–33^ Studies have demonstrated that low and very low molecular weight degradation products of HA may elicit various pro-inflammatory responses, such as a marked difference between 20 kDa and 50 kDa HA on the upregulation of TNF-α expression in keratinocytes,^34^ or macrophages that undergo phenotypic changes dependent on the molecular weight of HA that correspond to either the (1) pro-inflammatory response for very low molecular weight (digest and 5 kDa) HA or (2) pro-resolving response for high molecular weight (800 and 3000 kDa) HA.^33^ Thus while the **HAPI** solubility is expected to be the highest for smaller HA fragments, a compromise had to be made in degradation to avoid the generation of higher amounts of HA with molecular weights below 20–50 kDa.

Acidic degradation of commercial 1.6 MDa sodium salt in solution at pH 1 and 60 °C for 24 h, subsequent neutralization with TBA-OH and dialysis against deionized water to remove small HA fragments and excess TBA-Cl, afforded the TBA-salt of HA with a molecular weight of 50 kDa, which was converted to **HAPI** in the same fashion as the high molecular weight one.

### Photophysics

**Fig. 3:**
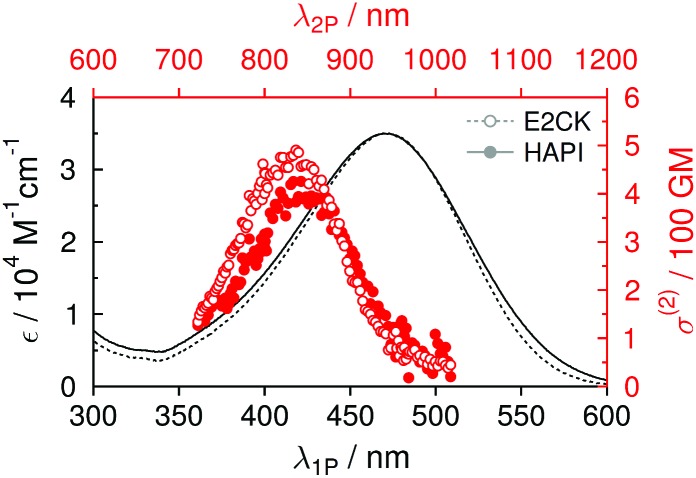
One-photon (black) and two-photon (red) absorption spectra of **HAPI** and **E2CK** in PBS.

The one-photon absorption (1PA) spectrum of **HAPI** resembles both the spectral bandshape (maximum around 470 nm) and the maximum extinction coefficient (approx. 35 × 10^3^ M^–1^ cm^–1^) of the reference 2PI (**E2CK**) almost to perfection (see Table 1 and Fig. 3). The lowest energy absorption band exhibits a small shoulder at higher energies (<400 nm), which, in analogy to previous findings,^16,17^ may be attributed to a one-photon forbidden (two-photon allowed) transition that has its origin in the excitonic interaction of the two branches of the D–π-A–π-D system.^35^ The fluorescence of **E2CK** and **HAPI** peaks around 650 nm, with fluorescence quantum yields in PBS being very low (around 0.2%). The associated fluorescence lifetimes are significantly below the time-resolution of our set-up (<100 ps). Whether this low emission efficiency and fast deactivation of the singlet state has its origin in an ultrafast internal conversion channel back to the ground state or an enhanced intersystem crossing channel to the triplet state is currently being investigated using ultrafast transient absorption spectroscopy.

**Table 1:** Basic photophysical properties in PBS at 20 °C. *λ*_abs_, wavelength of 1PA maximum. *ε*_max_, molar extinction coefficient at 1PA maximum. *φ*, fluorescence quantum yield

	*λ*_abs_ [nm]	*ε*_max_ [M^–1^ cm^–1^]	*λ*_2P_ [nm]	*σ* ^(2)^ [GM]	*λ*_em_ [nm]	*φ* [10^–3^]
**E2CK**	471	35 000	830	480	∼650	2.5
**HAPI**	466	35 000	840	400	∼650	1.8

Two-photon (2P) induced fluorescence was used to record the corresponding two-photon excitation spectra and cross sections. Again, **HAPI** shows almost identical behavior to **E2CK**, with the 2PA maximum being around 830–840 nm and maximum 2PA cross-section values in the range of approx. 400–500 GM (see Table 1 and Fig. 3). The slightly lower maximum 2PA cross section of **HAPI** compared to **E2CK** could be the result of slightly different degrees of planarity of the 2PI chromophores (different substituents on the amino-donor groups and on the central cyclohexanone ring) as has been previously reported for similar derivatives.^16^ However, it is worth noting that this observed difference is also in the same order of magnitude as the potential error related to the very low fluorescence quantum yields as well as the error related to the NMR-determination of **HAPI**'s DS, both of which affect the calculated 2P cross sections. The fact that the strong 2P transition shows up at lower wavelengths is in line with the above mentioned excitonic interaction of the two branches of the D–π-A–π-D system and has been extensively described^36,37^ and observed in the literature for structurally similar systems.^17^

### 2PI transmembrane migration

**Fig. 4:**
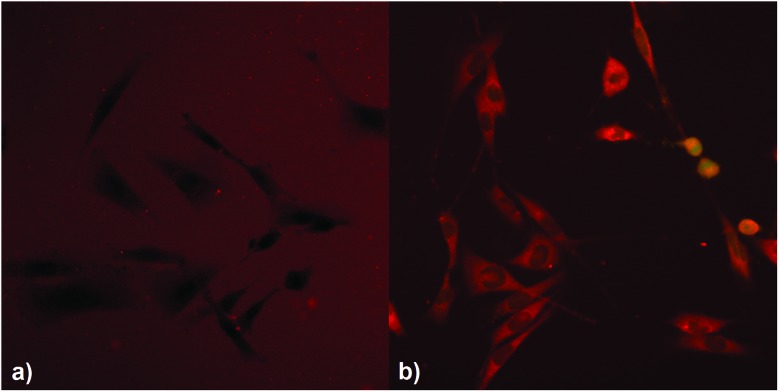
LSM images visualizing the autofluorescence of **HAPI** (a) and **E2CK** (b) around and into MC3T3 cells, indicating their different transmembrane migration behavior. The brightness of **HAPI**-image had to be digitally enhanced compared to **E2CK** images for better visibility of dark cells against a brighter fluorescence background.

To investigate the effect of the covalent linking of the 2PI to HA on transmembrane migration from the ECM to the cytosol, MC3T3 cells were exposed to solutions of either **HAPI** or **E2CK**. The distribution of the 2PIs was then visualized by laser scanning microscopy (LSM) imaging, taking advantage of the autofluorescence of the 2PI chromophores. **HAPI** does not readily enter the cells and exhibits a weak fluorescence (Fig. 4(a), brightness digitally enhanced) in the surrounding medium, the cells themselves appearing comparatively dark against this background fluorescence. In the case of **E2CK** the picture is reversed (see Fig. 4(b)), with the cells readily taking up the 2PI and various structures inside the cells appearing brightly stained. This provides a strong indication that the transmembrane migration of the 2PI is effectively hindered by covalent linkage to HA.

### Cytotoxicity assay

**Fig. 5:**
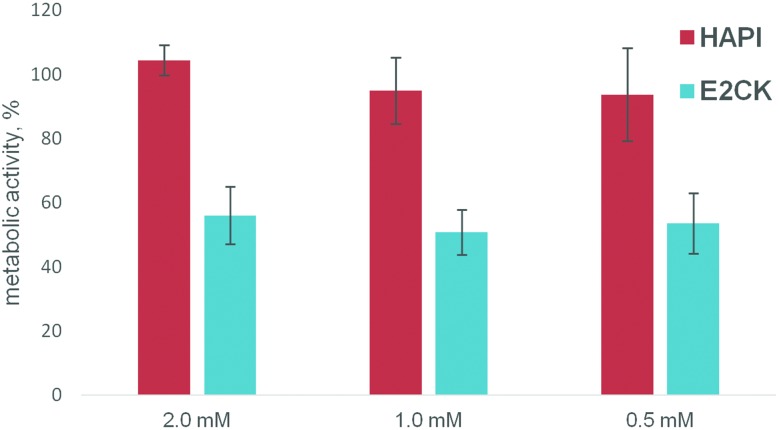
Influence of different concentrations of **HAPI** and **E2CK** on the metabolic activity of MC3T3-E1 cells after 5 hours (PrestoBlue cell viability test). All values are presented as % of the positive untreated control.

During the 2PP sample preparation and printing process, the cells come into prolonged contact with the material containing dissolved 2PI, even if they are destined to be trapped in the cavities of a structure and are thus not directly exposed to laser radiation and laser-induced radicals. Thus besides phototoxicity, the cytocompatibility of the 2PIs without excitation by 2PA is also of interest. MC3T3 cells were exposed to the αMEM cell culture medium containing either **HAPI** or the water-soluble 2PI reference **E2CK** ^6^ at various concentrations. After an exposure time of 5 hours, a representative time frame for the 2PP manufacturing even of relatively large structures, the metabolic activity of cells was evaluated with a PrestoBlue assay. While a comparison of **HAPI** with a control sample incubated under the same conditions but without 2PI showed no statistically significant difference, in the concentration range from 0.5 mM–2 mM the metabolic activity for **E2CK** was decreased *versus* the control by 40 ± 9% to 49 ± 9%, indicating a significant cytotoxicity of the reference compound but not **HAPI** (see Fig. 5).

### 2PP encapsulation of cells

To examine the functionality of the **HAPI** in its dedicated application of living cell encapsulation, 3D polymeric hydrogel structures were printed *via* 2PP in a cell suspension containing dissolved **HAPI** and GelMod.

**Fig. 6:**
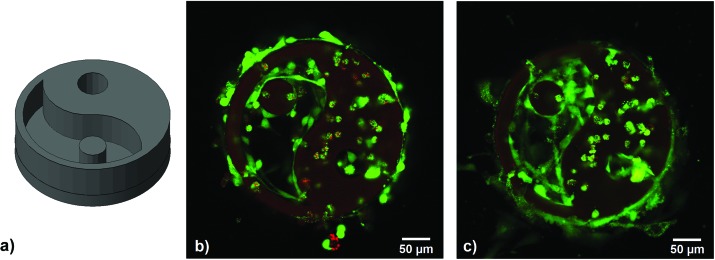
(a) CAD model of the yin–yang structure (upside down for better visibility of the cavities, top layer in the picture is joined to the glass substrate), (b) encapsulated MC3T3 24 h after 2PP structuring – cells in the cavity are stretching, encapsulated cells show a round morphology, (c) same structure 5 days after 2PP – cells in the cavity are proliferating, encapsulated cells are viable but round, possibly due to physical confinement.

While high molecular weight (1.6 MDa HA) **HAPI** was soluble in αMEM cell culture medium at the desired concentrations, upon mixing with GelMod solutions a phase separation occurred and **HAPI** formed a dense, deeply red gel-like precipitate, depriving the supernatant solution of the photoinitiator. An increase in the solubility was first attempted by using degraded lower molecular weight HA (50 kDa) as the starting material. This measure increased the compatibility significantly, but proved insufficient, so further stabilization of the solvation of the **HAPI** chains was attempted by adding methyl-β-cyclodextrin (MBCD), which is expected to form inclusion complexes with the apolar parts of the bound **MGABA**, thus weakening interactions like π–π stacking of the photoinitiator component. At a concentration of 10 mM MBCD was able to stabilize mixtures containing 15% GelMod and 1 mM low molecular weight **HAPI** so that 2PP structuring was successful. It should be noted that the literature^38^ suggests the cytotoxicity of MBCD at the required concentrations and our preliminary tests showed a greater reduction of the metabolic activity by 10 mM MBCD alone than by 2 mM **E2CK**. However in the case of actual 2PP encapsulation, cell survival was excellent (see Fig. 6) likely because MBCD predominantly binds to the large amount of GelMod present in the hydrogel precursor formulations, and thus does not interfere with the cells.

For 2PP encapsulation of live cells, a 3D yin–yang structure was written into a suspension of MC3T3 cells in αMEM cell culture medium containing 15% GelMod, 1 mM **HAPI** and 10 mM MBCD. The yin–yang structure (see Fig. 6) allows for an easy side-by-side comparison of cells that have been exposed to the laser and are encapsulated in the hydrogel and cells that have not been exposed but are trapped in the medium-filled cavity of the structure.^5^

Cell viability was assayed 24 h and 5 days after printing by live/dead-staining and LSM imaging, showing live cells in green and dead cells in red. The hydrogel itself also appears stained red due to the autofluorescence of residual 2PI. Throughout the examined period, cell survival was excellent, both in the polymerized parts and the cavities of the structure. After 5 days, the cells in the cavity had a stretched morphology and proliferated, while encapsulated cells were still alive but showed a round morphology, possibly due to physical confinement within the surrounding GelMod matrix (see Fig. 6).

## Conclusions

The two-photon initiator precursor **MGABA** was developed, containing a donor–π-acceptor–π-donor structure motif for efficient two-photon absorption and a sterically accessible primary amino group to allow for high degrees of substitution in subsequent modification reactions on hyaluronan. A novel hyaluronan-based polymeric two-photon initiator (**HAPI**) was prepared and characterized.

Laser scanning microscopy of cells incubated with either **HAPI** or the reference small molecule 2PI E2CK indicated that the macromolecular nature of **HAPI** indeed hinders 2PI transmembrane migration effectively.

The assay of cytotoxicity independent of two-photon excitation proved a superior biocompatibility of **HAPI** compared to the reference water-soluble two-photon initiator E2CK. 3D hydrogel structures containing living cells were successfully produced by the 2PP crosslinking of GelMod with **HAPI** in the presence of methyl-β-cyclodextrin as an additive to stabilize the hydrogel precursor solutions. The samples were followed up for at least 5 days using a live/dead-staining assay confirming the viability of the cells over this period. These results indicate the low phototoxicity and high efficiency of **HAPI**, as evidenced by a high scanning speed (100 mm s^–1^) during the 2PP process. While optimization of the solubility behavior and further investigation of the relationship between the structure of **HAPI** and two-photon initiation activity are desirable, the system shows excellent biocompatibility and is a promising basis for further developments of the encapsulation of live cells by two-photon induced polymerization.
