# Supplementary material for: A biocompatible macromolecular two-photon initiator based on hyaluronan
Source: Polym Chem. 2016 Nov 29;8(2):451–60. doi: 10.1039/c6py01787h (PMC5310395; doi:10.1039/c6py01787h)
Supplement: Supplementary file 1 [file PY-008-C6PY01787H-s001.pdf]

Electronic Supplementary Material (ESI) for Polymer Chemistry.
This journal is © The Royal Society of Chemistry 2016

Electronic Supplementary Information for

Biocompatible macromolecular two-photon initiator based on hyaluronan

(3E,5E)-N-[3,5-bis[[4-(dimethylamino)phenyl]methylene]-4-oxocyclohexyl]-carbamic acid 1,1-dimethylethyl ester (Boc-MCNK)

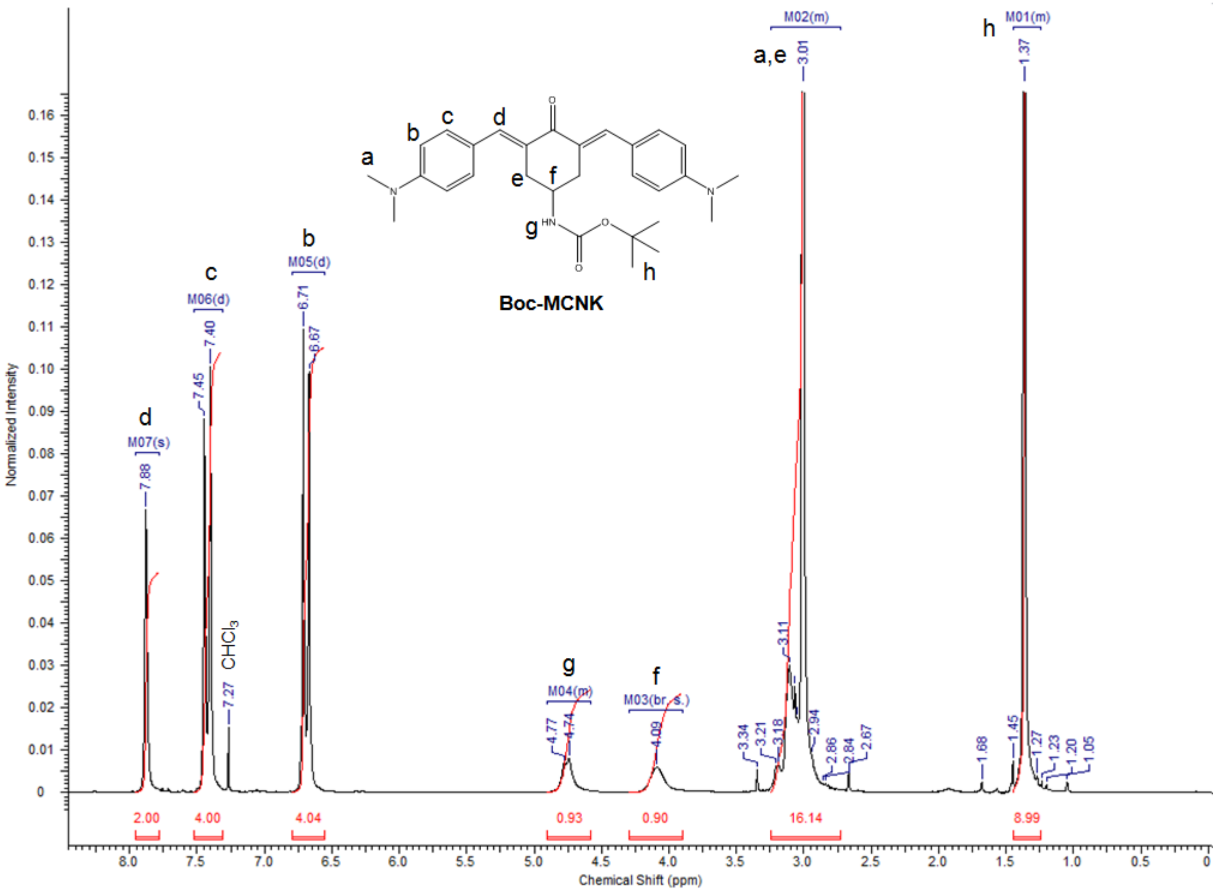

Fig. S1: ^{1}H NMR of **Boc-MCNK**

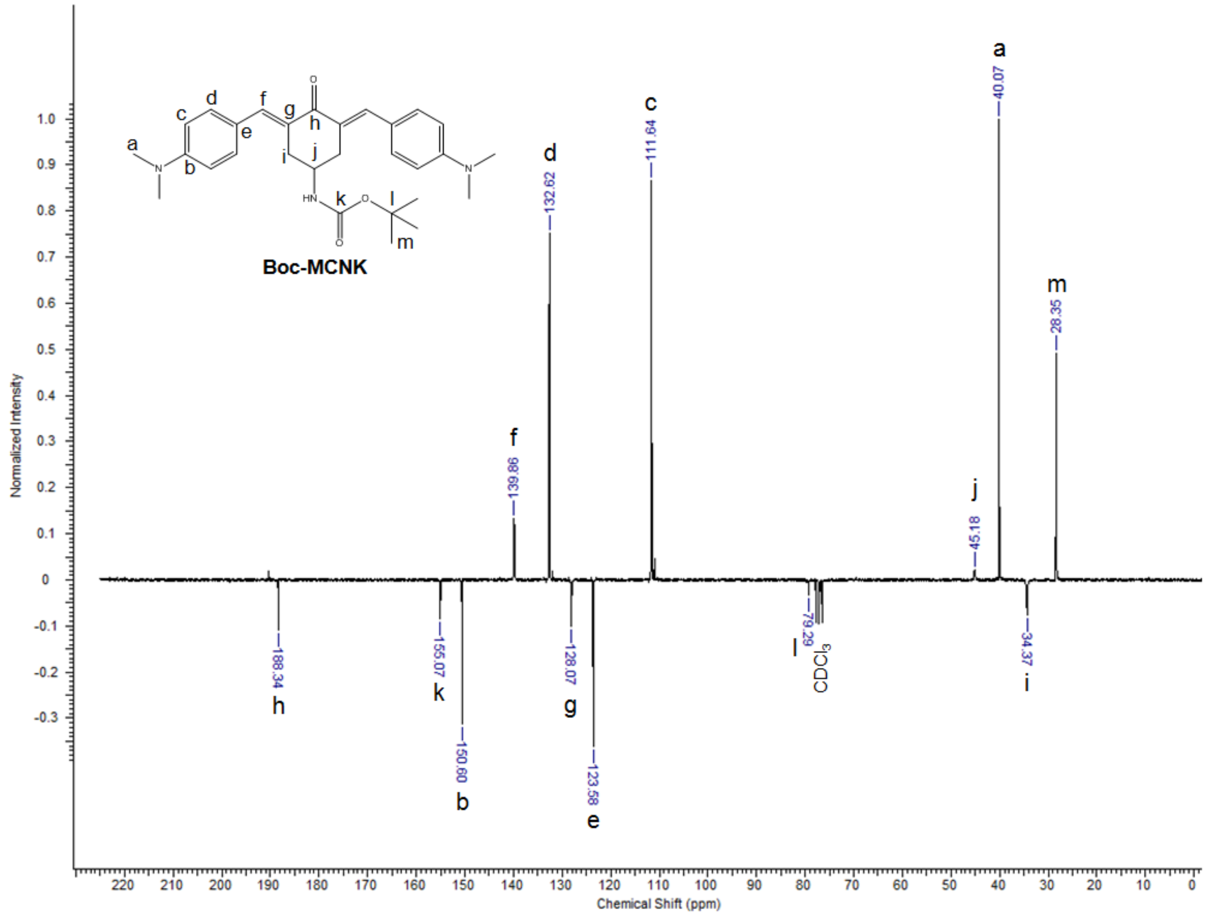

Fig. S2: ^{13}C APT NMR of **Boc-MCNK**

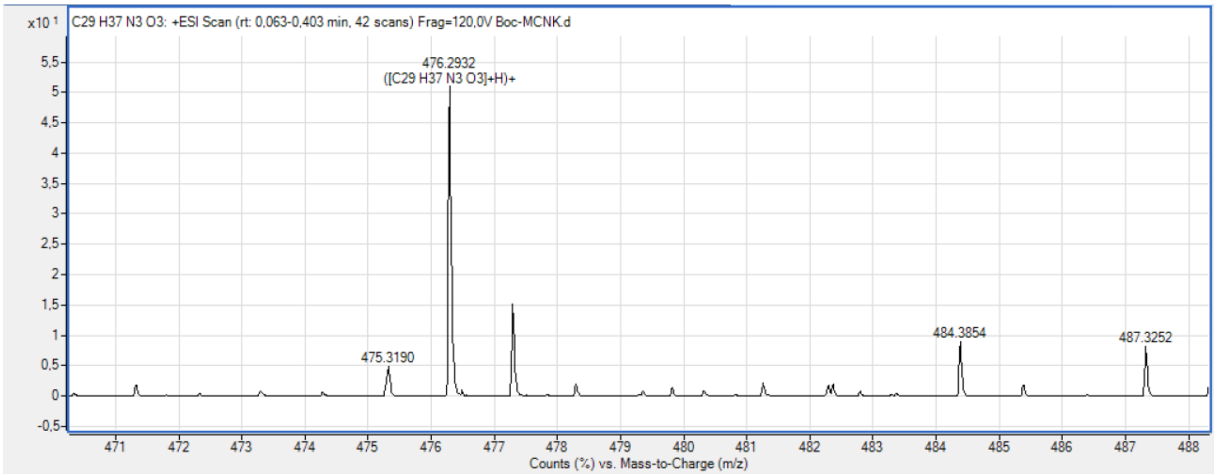

Fig. S3: HRMS showing [M+H]$^{+}$-peak of **Boc-MCNK**

(2*E*,6*E*)-4-amino-2,6-bis[[4-(dimethylamino)phenyl]methylene]cyclohexanone (MCNK)

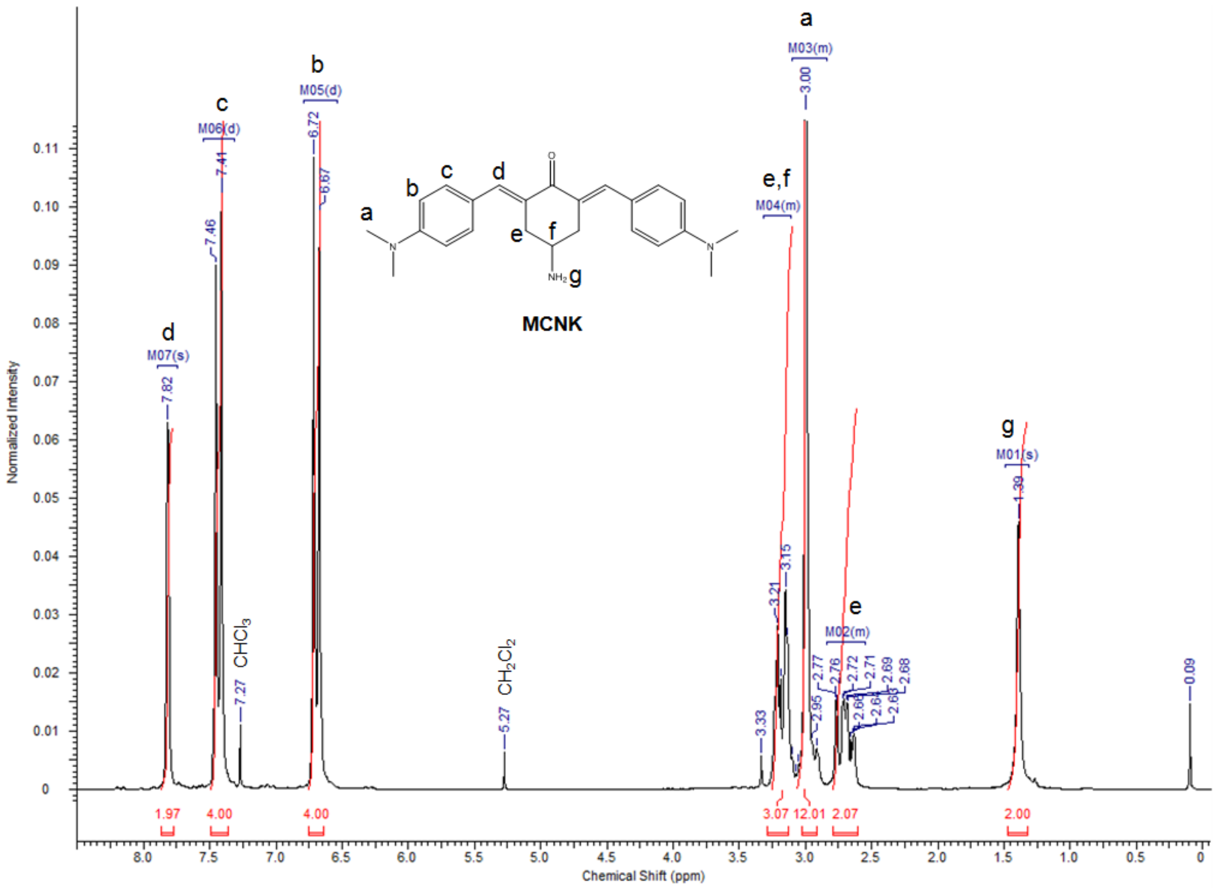

Fig. S4: ^{1}H NMR of **MCNK**

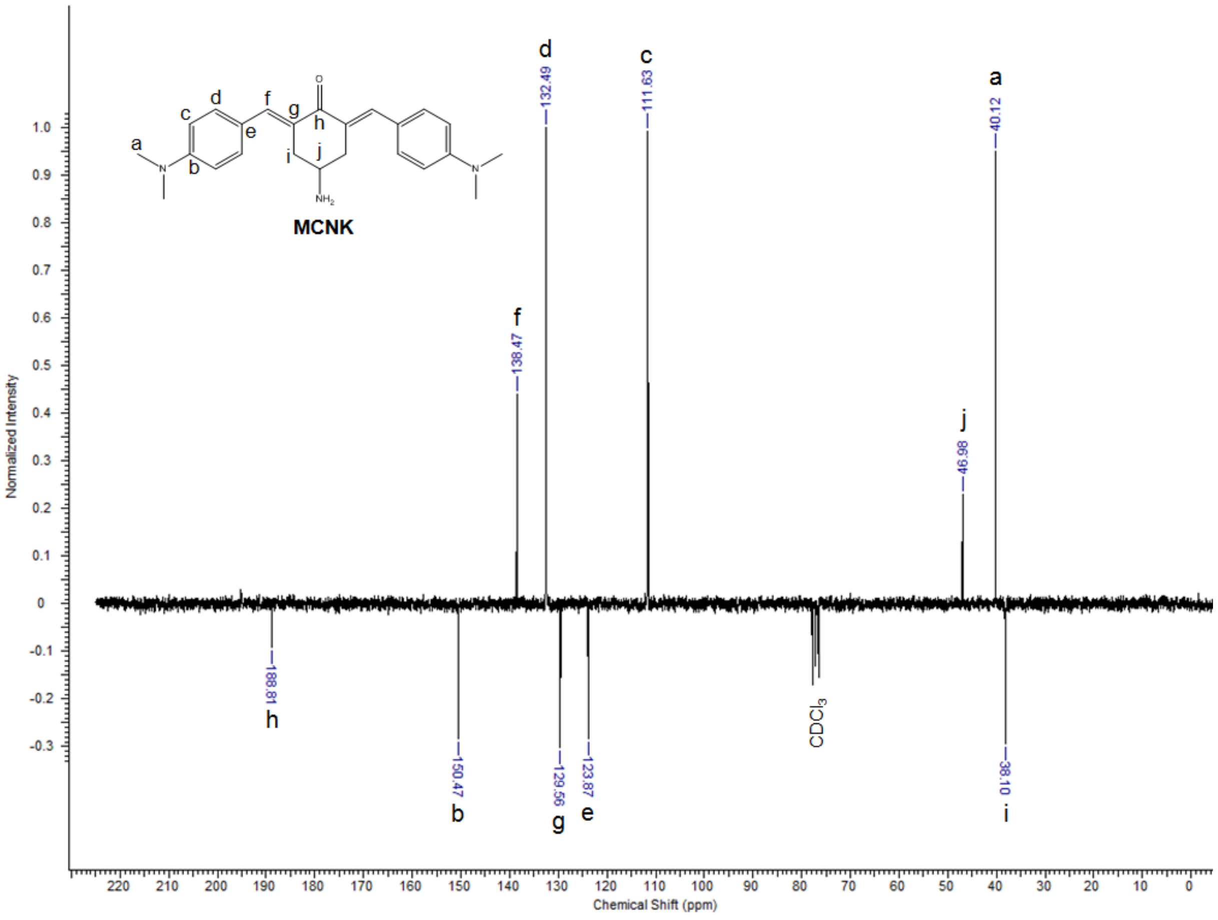

Fig. S5: ^{13}C APT NMR of **MCNK**

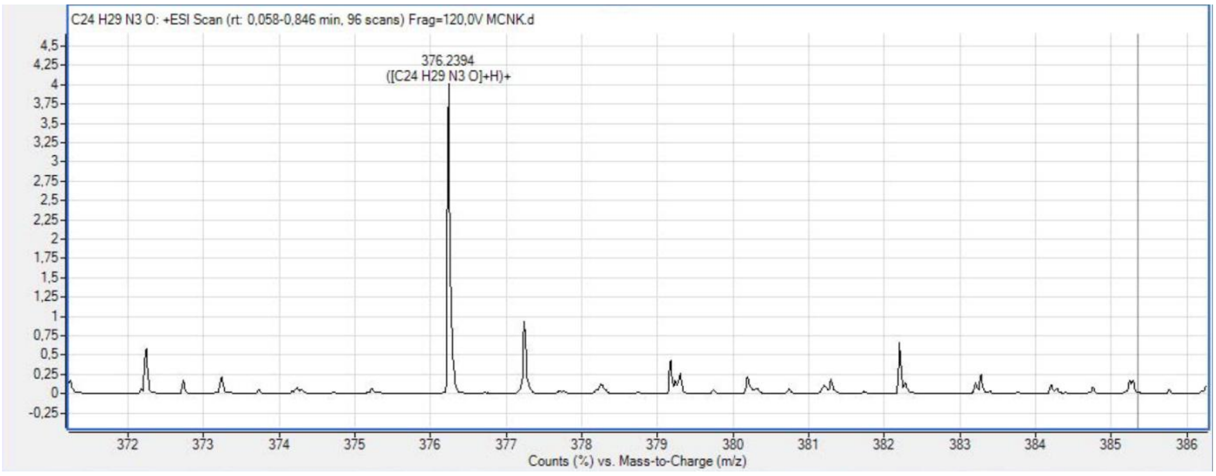

Fig. S6: HRMS showing $[M+H]^{+}$-peak of **MCNK**

(3E,5E)-N-[4-[[3,5-bis[[4-(dimethylamino)phenyl]methylene]-4-oxocyclohexyl]amino]-4-oxobutyl]carbamic acid 1,1-dimethylethyl ester (Boc-MGABA)

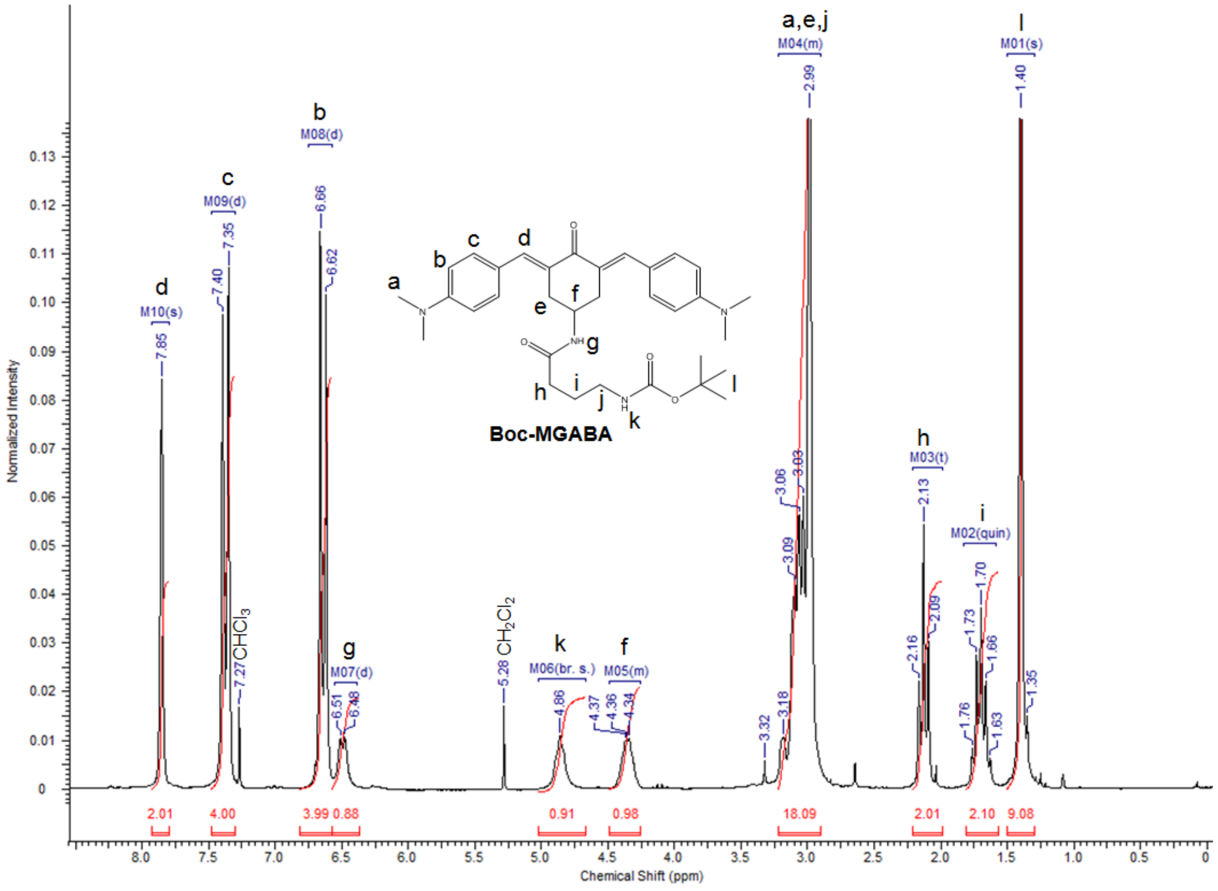

Fig. S7: ^{1}H NMR of **Boc-MGABA**

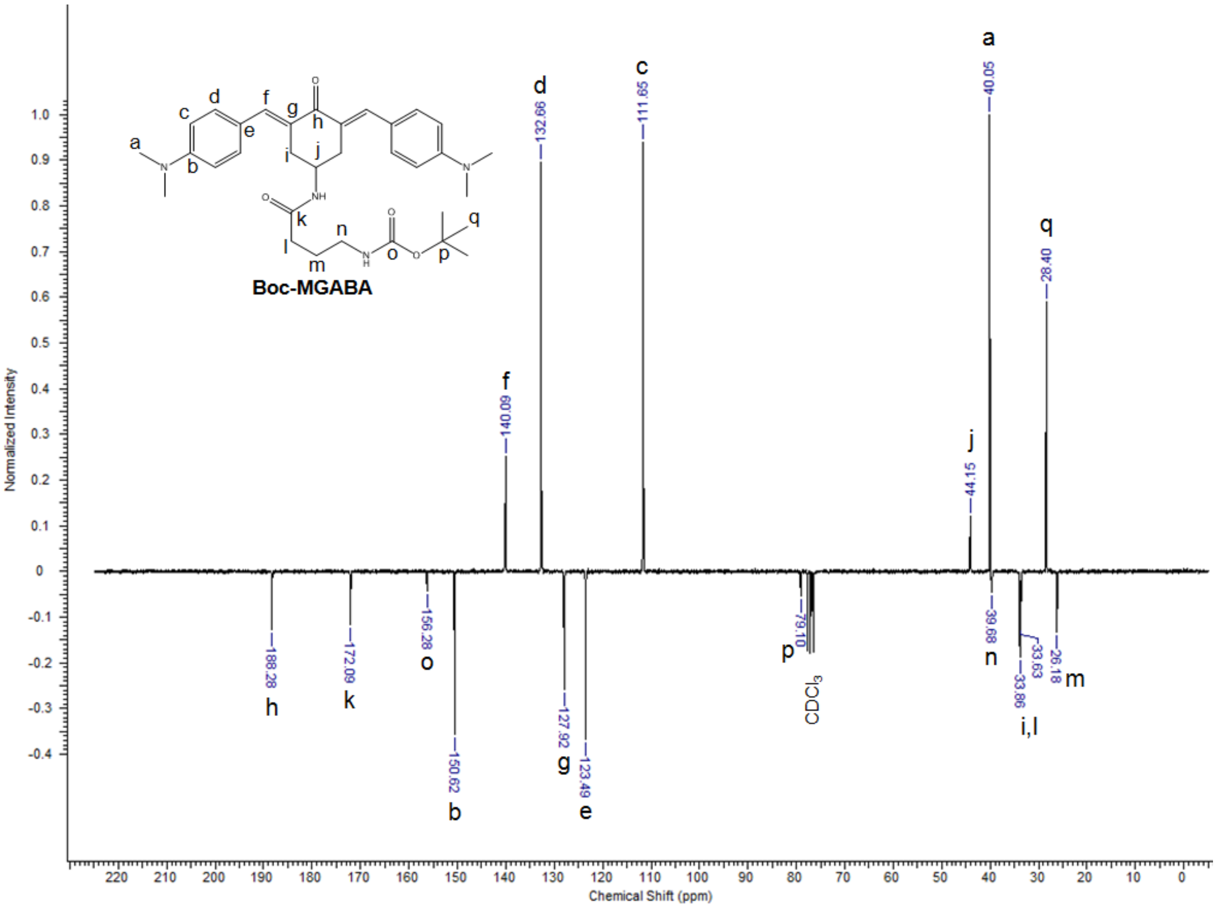

Fig. S8: ^{13}C NMR of **Boc-MGABA**

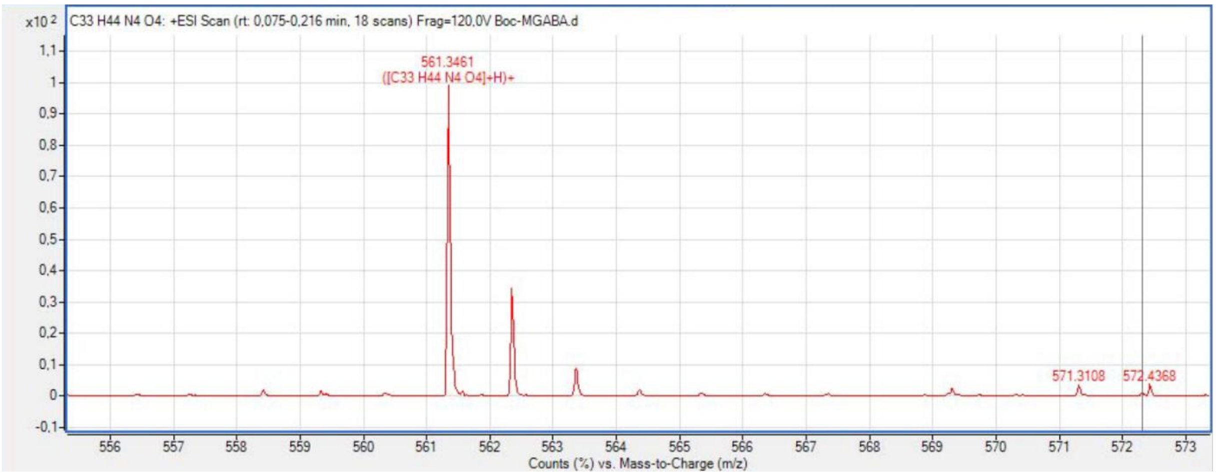

Fig. S9: HRMS showing $[M+H]^+$-peak of **Boc-MGABA**

(3E,5E)-4-amino-N-[3,5-bis[[4-(dimethylamino)phenyl]methylene]-4-oxocyclohexyl]butanamide (MGABA)

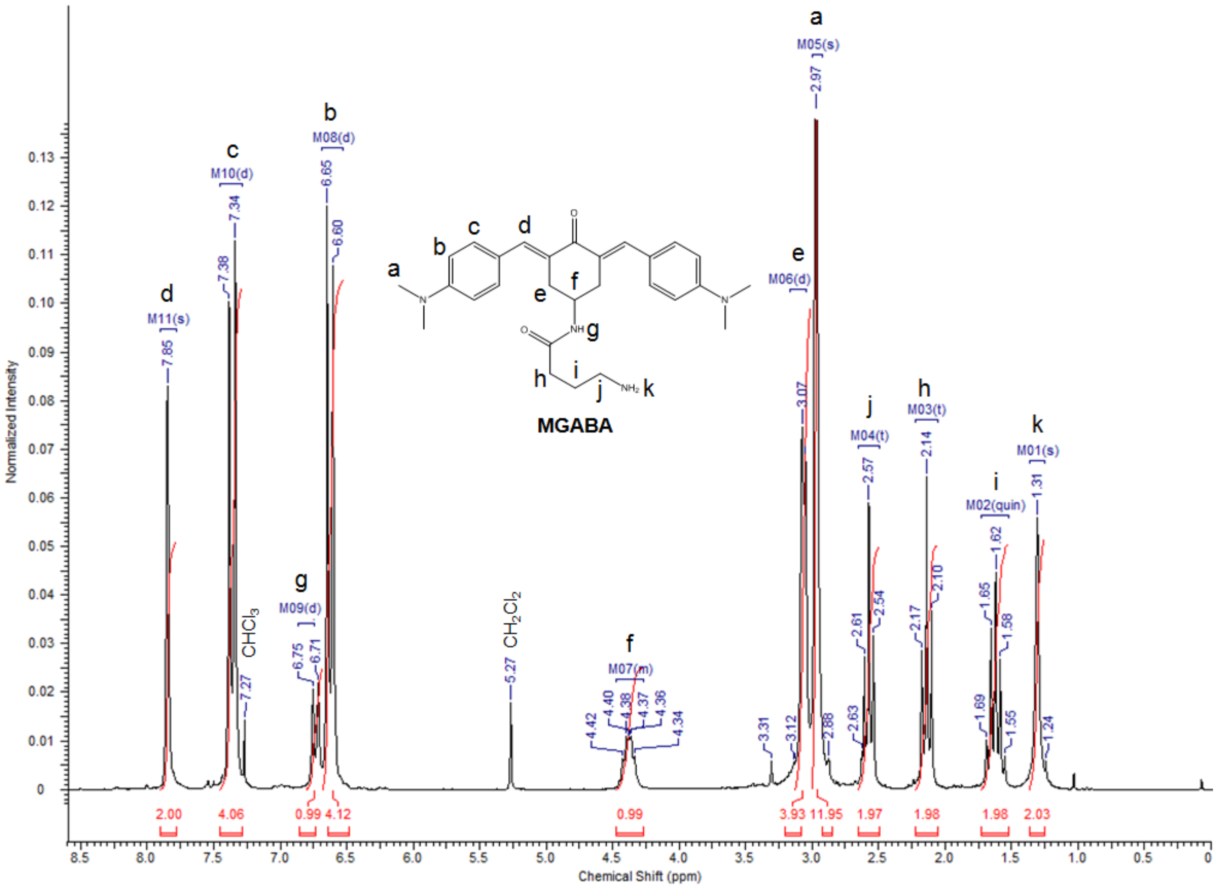

Fig. S10: ^{1}H NMR of **MGABA**

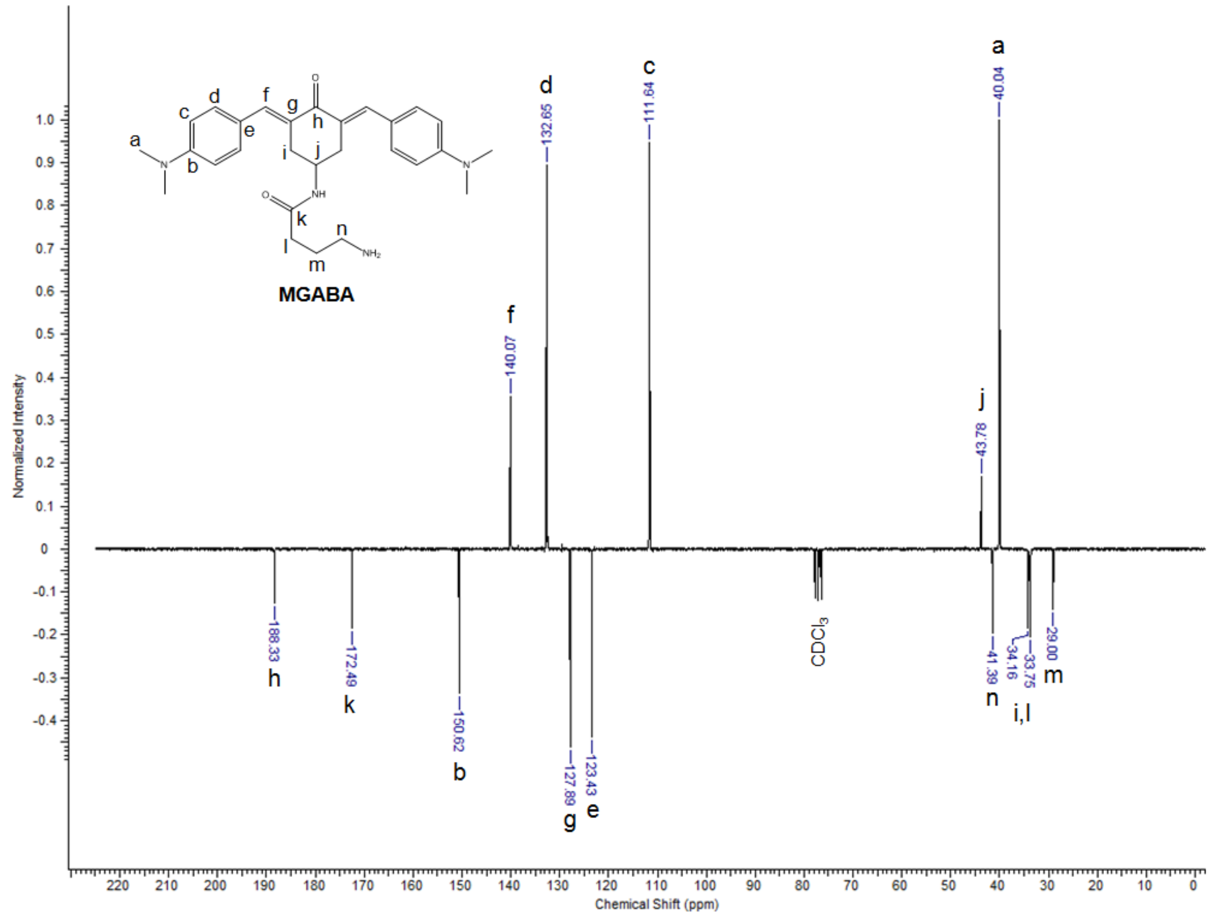

Fig. S11: ^{13}C APT NMR of **MGABA**

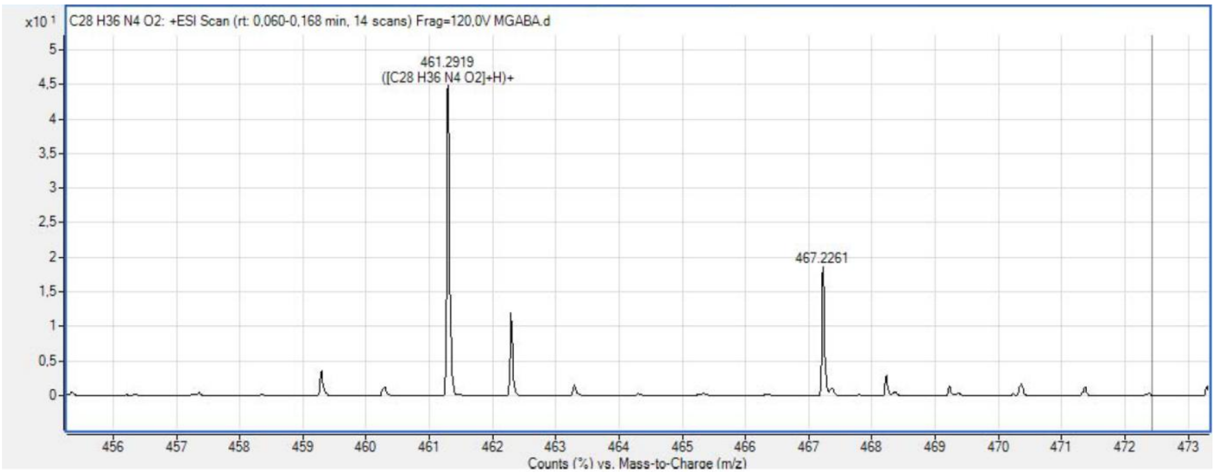

Fig. S12: HRMS showing $[M+H]^+$-peak of **MGABA**

Hyaluronan-based photoinitiator (HAPI)

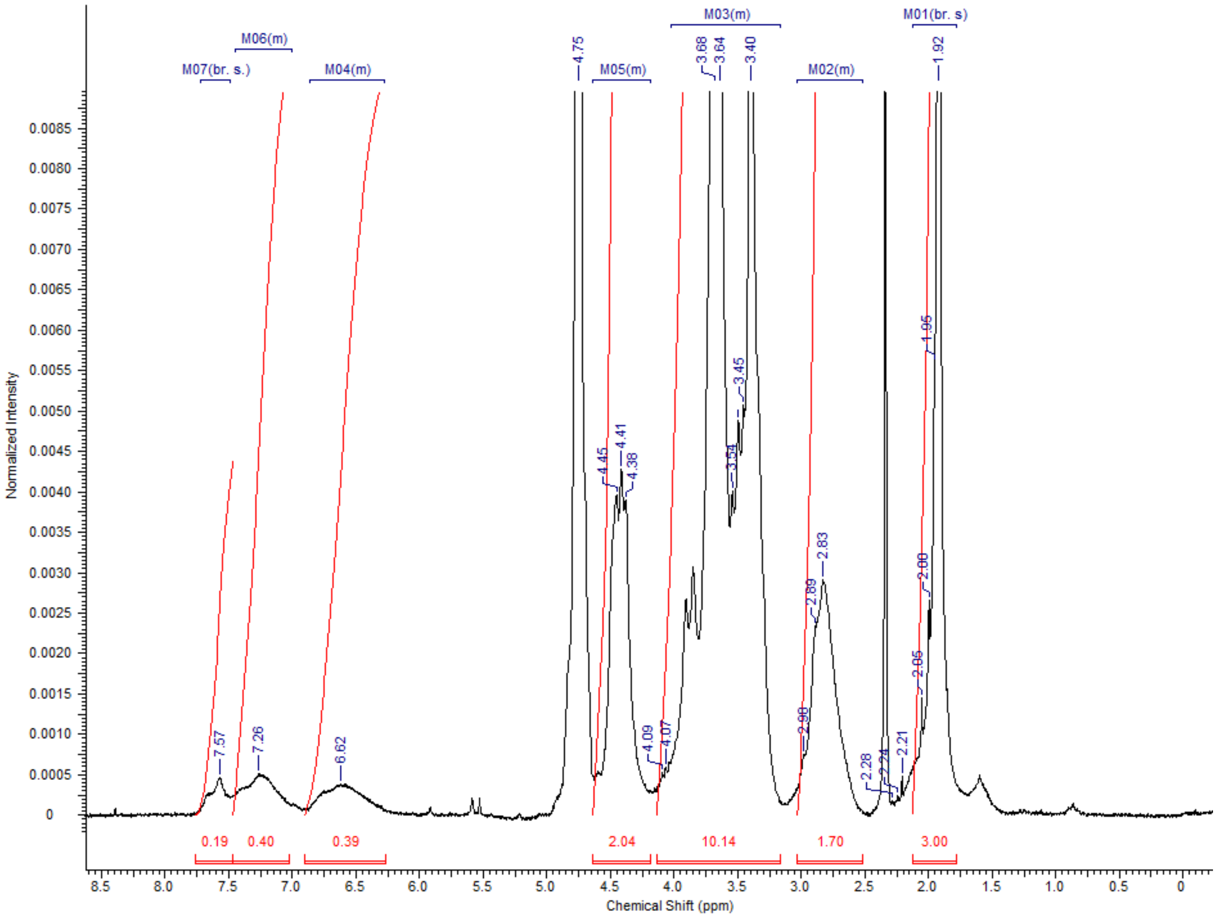

Fig. S13: ^{1}H NMR of **HAPI**

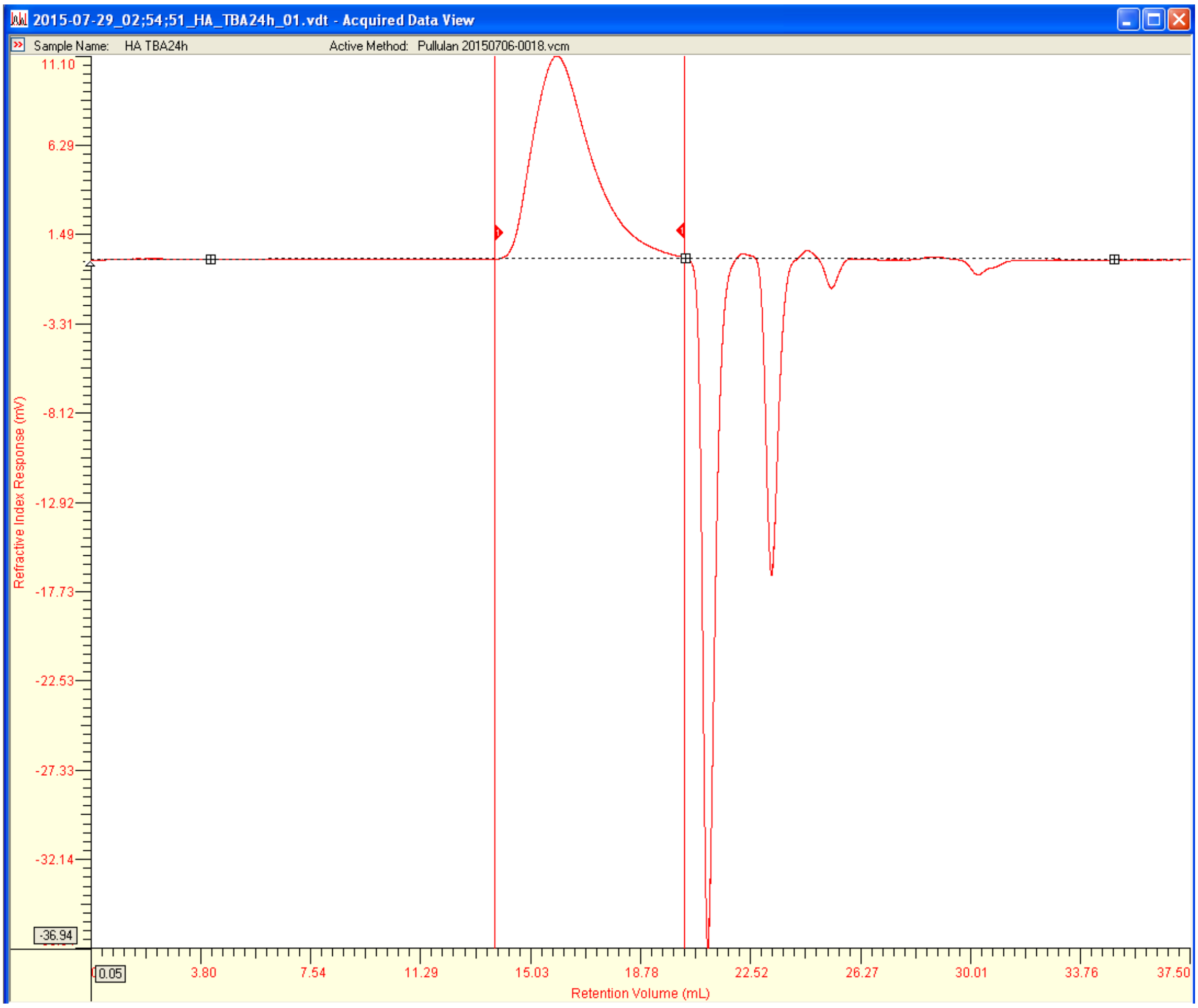

Fig. S14: GPC data of hydrolysed hyaluronan used in synthesis of **HAPI**

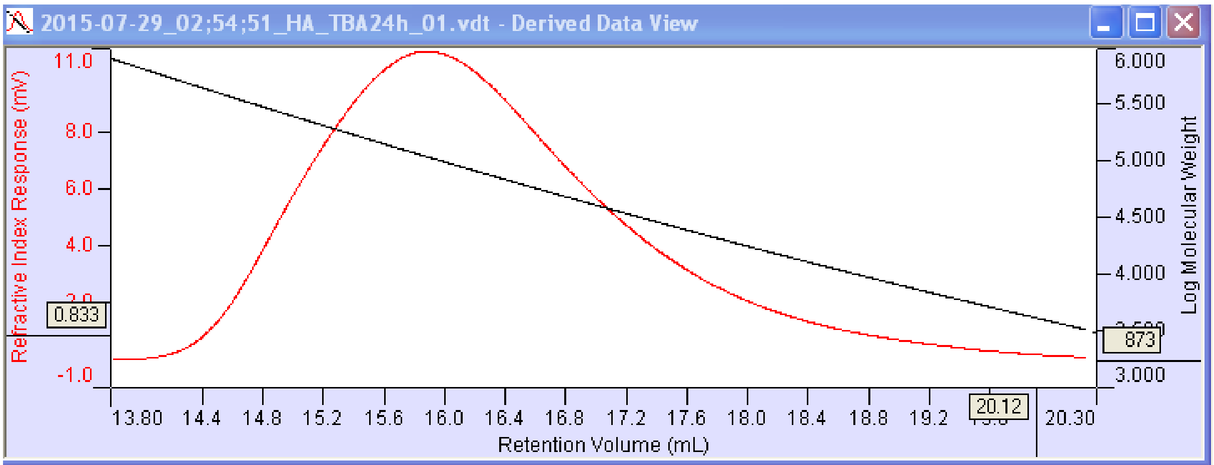

2015-07-29_02;54;51_HA_TBA24h_01.vdt - Result View

Conventional Calibration - Homopolymers : Results

Peak RV - (ml)	15.883
Mn - (Daltons)	50,039
Mw - (Daltons)	110,082
Mz - (Daltons)	177,861
Mp - (Daltons)	108,425
Mw / Mn	2.200
Percent Above Mw: 0	0.000
Percent Below Mw: 0	0.000
Mw 10.0% Low	14,659
Mw 10.0% High	296,835
RI Area - (mvml)	24.80
UV Area - (mvml)	0.00

Annotation	
Method File	Pullulan 20150706-0018.vcm
Limits File	_TBA24h_01-Pullulan 20150706-0018-0002.lim
Date Acquired	Jul 29, 2015 - 02:54:51
Solvent	BPS 5mM pH7.3 NaCl 0.15M
Acquisition Operator	admin : Administrator
Calculation Operator	autologin : autologin
Column Set	ultrahydrogel linear
System	Ultrahydrogel linear
Flow Rate - (ml/min)	0.500
Inj Volume - (ul)	160.0
Volume Increment - (ml)	0.00417
Detector Temp. - (deg C)	35.0
Column Temp. - (deg C)	30.0
OmniSEC Build Number	268

Fig. S15: GPC data of hydrolysed hyaluronan used in synthesis of **HAPI**
